# Association between atmospheric pollutant levels and oxidative stress in pregnant women and newborns in Urumqi

**DOI:** 10.1186/s12889-023-15222-9

**Published:** 2023-02-14

**Authors:** Linghui Zhu, Ying Lu, Fang Cheng, Luyi Zhang, Aliya Yusan, Xialidan Alifu, Ayixiemuguli Alimasi, Xiahaida Alemujiang

**Affiliations:** 1 School of Public Health, Xinjiang Medical University, 830054 Urumqi, China; 2 International School of Public Health and One Health, Hainan Medical University, 571199 Haikou, China; 3 Department of Obstetrics, Fifth Affiliated Hospital, Xinjiang Medical University, Urumqi, Xinjiang China; 4 China Energy Construction Group Xinjiang Electric Power Design Institute Co., Ltd. Testing Center, 830054 Urumqi, Xinjiang China; 5 Department of Epidemiology and Health Statistics, School of Public Health, Zhejiang University, Zhejiang, China; 6 State Key Laboratory of Pathogenesis, Prevention and Treatment of High Incidence Diseases in Central Asia, 830011 Urumqi, Xinjiang China; 7 The First People’s Hospital of Urumqi (Urumqi Children’s Hospital), 830000 Urumqi, China; 8 Human resource department, People’s Hospital of Xinjiang Uygur Autonomous Region, Tianshan District No. 91 Tianchi Road, 830001 Urumqi, Xinjiang China

**Keywords:** Pregnant women, Polycyclic aromatic hydrocarbons, Deoxyguanosine, Air pollution, Oxidative stress, Newborn infant

## Abstract

**Background:**

Frequent heavy air pollution occurred during the winter heating season of northern China. Particulate air pollution is a serious concern in Urumqi during heating season. Exposure to air pollution is known to increase adverse health outcomes, particularly oxidative damage. This study aimed to evaluate the impact of air pollution on oxidative damage around pregnant women and newborns in Urumqi.

**Methods:**

This prospective observational study enrolled pregnant women in the Fifth Affiliated Hospital of Xinjiang Medical University between January 2019 and October 2019. Pregnant women and newborns were allocated into a heating season group (January - end of April 2019, October 2019) or non-heating season group (June 2019 - end of September) according to the specific delivery time. Venous blood, urine from the women and cord blood from their newborns were collected to measure the levels of PAHs and 8-deoxyguanosine (8-OHdG), a measure of oxidative stress.

**Results:**

A total of 200 pregnant women and newborns were enrolled, with 100 pregnant women and newborns in the heating season group. Compared to the non-heating season group, the total contents of 8-OHdG in maternal urine, PAHs and 8-OHdG in maternal plasma and neonatal cord blood were higher in the heating season group (all P < 0.001). The average values for AQI, PM_2.5_, PM_10_, SO_2_, NO_2_, and CO were higher in the heating season group (all P < 0.001). Maternal and neonatal PAHs were correlated with 8-OHdG measurements in maternal urine (r = 0.288, P < 0.001 and r = 0.336, P < 0.001) and neonatal umbilical cord blood (r = 0.296, P < 0.001 and r = 0.252, P < 0.001). There was also a positive relationship between PAHs, 8-OHdG levels in pregnant women and their newborns and proximate air pollutant concentrations (all P *<* 0.05). Based on the results of multiple linear regression analysis, it was found that air pollutants(PM_10_, 0_3_) had a great influence on the level of 8-OHdG in neonatal cord blood, and the contribution rate was high(R^2^ = 0.320). Based on the epidemiological questionnaire, a multiple linear regression model was established(R^2^ = 0.496). We found that 8-OHdG levels in neonatal umbilical cord blood were mainly affected by two aspects: (1) Biological samples collected during heating had higher levels of 8-OHdG in neonatal umbilical cord blood. (2) Study may suggest that in neonates, males are more sensitive to oxidative damage.

**Conclusion:**

Particulate air pollution may increase PAHs exposure and oxidative DNA damage in pregnant women and newborns.

## Background

Urumqi is a city in Northwest China with cold weather in winter and spring, necessitating the use of district heating for up to six months a year. Urumqi is burdened with high levels of atmospheric particulate matter compared to other cities in China [1]. According to the air quality reports collected over the past 10 years, Urumqi has more severe air pollution during the central heating season from late October to mid-April compared to the non-heating season from late April to early October [1–4].

Polycyclic aromatic hydrocarbons (PAHs) are known carcinogens present in atmospheric particulate matter [5, 6]. PAHs are created from the incomplete combustion of fuels including wood, oil, coal, and gas [7]. Most pollutants exert their biological toxicity by inducing the oxidation of DNA [8–10]. Accordingly, PAHs produce a large amount of reactive oxygen species during metabolism, which attack DNA molecules to produce oxidative DNA damage. Epidemiological studies have shown that there is a close relationship between PAHs exposure and DNA oxidative damage [11].

Pregnant women are at an increased risk of adverse outcomes from PAHs exposure. Many follow-up studies have found that exposure to PAHs during the fetal period increases the risk of abnormal physical development including low birth weight and congenital defects [12–15]. It also has adverse effects on fetal neural development, cognitive development, and can cause behavior problems [16–18]. After birth, PAHs can be transmitted through breast milk and further impact newborns [19]. Therefore, it is imperative to fully understand the relationship between particulate matter exposure, the concentration of PAHs, and the level of DNA oxidative damage in pregnant women and their newborns.

In this study, pregnant women and their newborns were divided into a heating season group and a non-heating season group to explore the impact of air pollution on oxidative damage around birth.

## Methods

### Study design and participants

This prospective observational study included pregnant women seen by the Fifth Affiliated Hospital of Xinjiang Medical University between January 2019 and October 2019. Pregnant women and newborns were allocated into a heating season group (January 2019 - end of April, October 2019) or non-heating season group (June - end of September 2019) according to the specific delivery time and the date of birth.

The inclusion criteria were: (1) age ≥ 18 years old; (2) the pregnant woman gave birth at term and had a singleton live; (3) Permanent residents of Urumqi or residents who have lived in Urumqi for more than 1 year; (4) Individuals who were able and willing to provide contact information and biological samples. The exclusion criteria were: (1) diagnosis of diabetes mellitus, hypertension, or other acute and chronic diseases; (2) history of genetic or infectious disease; (3) history of smoking or drinking; (4) history of abnormal delivery; (5) use of medication abortion; (6) premature delivery. This study was approved by the Ethics Committee of Xinjiang Medical University (No.20190226-30). Written informed consent was obtained from the patients.

### Data collection and definition

#### Epidemiological questionnaire survey

The prospective study collected basic demographic information from patient medical records including age, nationality, and the date of delivery.In addition, we designed an epidemiological questionnaire including factors affecting the exposure level of PAHs in pregnant women (maternal age; Education for pregnant women; Number of deliveries by pregnant women; Biological sample collection period; Whether the housing has been renovated within one year before childbirth; The ventilation of the house during pregnancy; The distance of the residence from the road; Heating mode during pregnancy; Whether there is a tapestry in the housing during pregnancy; Eating barbecue and fried food during pregnancy; The husband smokes; Sex of newborn delivered), to explore its effect on 8-OHdG levels in neonatal cord blood.

Multiple linear regression model was established based on epidemiological questionnaire survey. The factors influencing PAHs exposure level of pregnant women were included in the study to explore the effect of PAHs on neonatal cord blood 8-OHdG level. (Table 1).

**Table 1:** The influencing factor assignment table

	Index	Assignment
X_1_	Pregnant women’s age	1 = 18- 2 = 25- 3 = 35-
X_2_	Pregnant women’s degree	1 = Primary school and below 2 = Middle school 3 = College or university
X_3_	Number of delivery of pregnant women (including this time)	1 = 1 2 = 2 3 = 3
X_4_	Biological sample collection period	1 = Non -heating period 2 = Heating period
X_5_	Whether the housing is decorated within one year before childbirth	1 = No 2 = Yes
X_6_	The ventilation of the house where the house lives during pregnancy	1 = Very good 2 = Good 3 = Poor
X_7_	The distance between the place of residence is from the road	1 = Far away(≥800 m)2 = Closer (400-800 m) 3 = Beside the road (≤400 m)
X_8_	Pregnancy heating method	1 = Heating 2 = Stove 3 = Electricity
X_9_	Whether there are tapestry in housing during pregnancy	1 = No 2 = Yes
X_10_	Eat barbecue and fried food during pregnancy	1 = No 2 = Yes
X_11_	Husband smoke	1 = No 2 = Yes
X_12_	Gender of Newborn	1 = Male 2 = Female

#### Air quality data collection

The real-time air quality data (PM_2.5_, PM_10_, SO_2_, NO_2,_ CO, O_3_, AQI) in Urumqi used in this study are from the authoritative data released by the Ministry of Ecology and Environment of the People’s Republic of China. We use the relevant programs of the Python crawler tool to quickly Extract the atmospheric air quality data information of Urumqi.

### Biological sample collection and detection

#### Biological sample collection

All biological samples were collected during delivery preparation on the day of delivery. Maternal venous blood samples (5 mL) were collected using ethylenediaminetetraacetic acid (EDTA) anticoagulant vacutainers. Neonatal umbilical cord blood (10 mL) was collected using EDTA anticoagulant vacutainers and ordinary blood collection vessels. Pregnant women were instructed to use a urine cup to collect middle segment urine (40 mL). The blood samples were centrifuged, and the serum was frozen at -80 ℃. Urine samples were frozen at -80 ℃ after sub packaging.

#### Detection of PAHs

The sample pretreatment included removing serum protein by hydrolysis with sodium hydroxide and ethanol water, performing liquid-liquid extraction with 3 mL of n-hexane for 3 times, collecting the organic phase and blowing it dry with nitrogen, making up the volume with methanol, filtering the membrane and loading it into a sample bottle for testing.

Then the concentration of PAHs (ug/L) in serum was measured by gas chromatography-mass spectrometry (GC-MS) (gas chromatograph: Agilent 7890 A; mass spectrometer: Waters, Waters Quattro Micro GC).

**Fig. 1:**
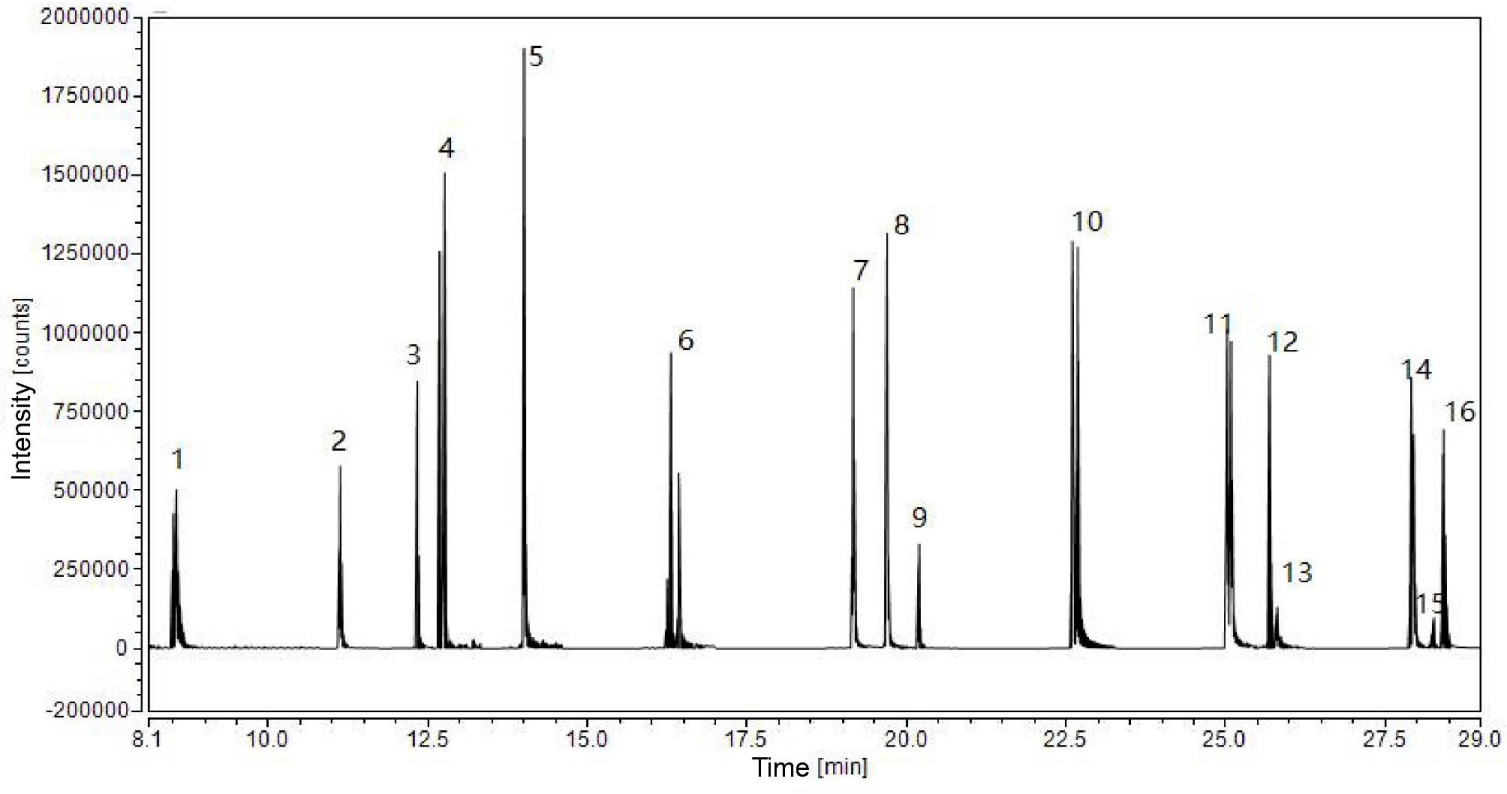
Total ion chromatograms of 16 kinds of PAHs. 1: Naphthalene; 2: Acenaphthylene; 3: Acenaphthene; 4: Fluorene; 5: Phenanthrene; 6: Anthracene; 7: Fluoranthene; 8: Pyrene; 9: Benzo[a]pyrene; 10: Chrysene; 11: Benzo [g, h, i] peryleme; 12: Benzo[a]anthracene; 13: Benzo [b] fluaranthene; 14: Benzo [k] fluaranthene; 15: Indeno [1,2,3-cd] pyrene; 16: Dibenz [a, h] anthracene

The 16 mixed standards required in the experiment were purchased from Tianjin Alta Technology Co., Ltd., the product number is 1ST4360-200 A, and the solvent is acetonitrile. The purity is 98%~99.9%, and the content of each substance is 200 µg/mL.Total concentrations of PAHs in each sample were obtained by adding concentrations of 16 PAHs. The 16 PAHs included naphthalene, acenaphthylene, acenaphthene, fluorene, phenanthrene, anthracene, fluoranthene, pyrene, benzo [a] pyrene, chrysene, benzo [g,h,i] peryleme, benzo [a] anthracene, benzo [b] fluaranthene, benzo [k] fluaranthene, indeno [1,2,3-cd] pyrene, dibenz [a, h] anthracene (Fig. 1).

Chromatographic conditions: DB-5MS capillary column (20 mm×0.18 m×0.18 μm); inlet temperature: 280 °C, splitless; injection volume: 1.0µL, column flow: 1.0mL/min (constant flow); column Temperature: hold at 80 °C for 2 min; rise to 180 °C at a rate of 20 °C/min, hold for 5 min; then rise to 280 °C at a rate of 10 °C/min, hold for 5 min. Carrier gas: high-purity helium, flow rate 0.7mL/min; injection mode: pulse splitless injection mode; injection volume: 0.5µL; purge time: 0.4 min.

Mass spectrometry conditions: ion bombardment source (EI); ion source temperature: 150 °C; ionization energy: 70 eV; interface temperature: 280 °C; quadrupole temperature: 150 °C;Solvent delay time: 5 min; scan mode: select MRM (multiple reaction monitoring) mode.

Sixteen kinds of PAHs and their minimum detection limits was Naphthalene (0.13ng/mL), Acenaphthylene (0.43ng/mL), Acenaphthene (0.12ng/mL), Fluorene (0.02ng/mL), Phenanthrene (0.03ng/mL), Anthracene(0.05ng/mL), Fluoranthene (0.02ng/mL), Pyrene (0.03ng/mL), Benzo[a]pyrene (0.42ng/mL), Chrysene (0.04ng/mL), Benzo[g,h,i]peryleme (0.21ng/mL), Benzo[a]anthracene (0.02ng/mL), Benzo[b]fluaranthene (0.11ng/mL), Benzo[k]fluaranthene (0.31ng/mL), Dibenz[a, h]anthracene (0.02ng/mL), Indeno[1,2,3-cd]pyren (0.20ng/mL). The spiked recoveries of the high, medium and low concentration groups of 16 target PAHs were 89.7-113.7%, 82.4-99.4%, 71.4-103.4%, respectively and the corresponding relative standard deviations (n = 3) were 7.2%~13.4%, 6.1%~12.4%, 4.8%~14.2%, respectively. The samples were measured three times in a row on the same day, and the intra-day precision was 1.5–8.9%.

#### Detection of 8-OHdG

Enzyme-linked immunosorbent assay (ELISA) was used to determine the 8-OHdG content of maternal venous blood, urine and neonatal umbilical cord blood. The process was carried out in strict accordance with the kit operating instruction, including thawing, sample addition, incubation, solution preparation, washing, color development, termination, and OD (optical density) determination. The abscissa is the OD-value and the ordinate is the concentration of the standard. A standard curve was used to calculate the actual concentration of the sample.

### Exposure Assessment

This study carried out exposure assessment from two perspectives. (1) Due to the difficulty in implementing individualized exposure level monitoring, we used big data to reverse the entire gestational week of pregnancy according to their delivery date and gestational days, and collected data from each pregnant woman during pregnancy. The concentration of air pollutants experienced in one day was calculated as the average value of various pollutant concentrations of each pregnant woman during the entire pregnancy. According to the grouping, the average pollutant concentration of each group of 100 pregnant women was calculated separately; (2) Collect the blood, urine and cord blood of pregnant women and conduct relevant laboratory tests. The PAHs levels in the blood, urine and cord blood of pregnant women are taken as their actual exposure levels; The level of 8-OHdG was used as a reflection of the degree of oxidative damage.

The duration of exposure in this study was the entire pregnancy of each pregnant woman.

### Quality control

1. Mass spectrometry performance check: Before each GC-MS analysis, the mass spectrometer should be auto-tuned, and then the gas chromatograph and mass spectrometer should be set to the analysis method and the required instrument operating conditions, and be in a standby state. Directly inject 1.0 µL of n-hexane, run the method, and when the n-hexane peaks, the ion abundance of the mass fragments should meet the requirements, the baseline should be stable, and there will be no impurity peaks. Otherwise, the mass spectrometer ion source must be cleaned.
2. GC-MS blank test: Each batch of samples (no more than 20 samples) must run a blank test, and the target concentration from the results should not exceed the method detection limit. Otherwise, reagent blanks, instrument systems, and pre-processing procedures should be checked.
3. GC-MS parallel test: one pair of parallel samples should be analyzed for each batch of samples (up to 20 samples), and the relative deviation of the structure of parallel samples should be less than 30%.
4. GC-MS standard curve: the relative standard deviation of the target compound relative response factor in the standard curve is less than or equal to 20%. Otherwise, maintain the inlet. During continuous analysis, the intermediate concentration point of the calibration curve is analyzed every 24 h, and the relative standard deviation of the measurement result and the actual concentration value should be less than or equal to 20%. Otherwise, the standard curve must be redrawn.
5. In the questionnaire survey stage, the survey questionnaires prepared by the research group were used, and the investigators were strictly trained before the survey to ensure the uniformity of survey standards and content, and face-to-face surveys were adopted. The completed questionnaires will be compounded in time, filled in in time if there are any questions, and the qualified questionnaires will be numbered. During data entry, further verify the quality of the questionnaire, check the coding, avoid repetition and omission, and carry out logical error detection at the same time. The data adopts the double entry mode to check the consistency of data entry.

### Statistical analysis

Continuous variables with a normal distribution were described as mean ± standard deviation (SD). Categorical variables were presented as number. Comparisons for continuous data were performed using a student’s t-test or one‐way ANOVA. Categorical variables were compared using the chi‐square test or Fisher exact test. The 8-OHdG in maternal plasma, 8-OHdG in maternal urine, 8-OHdG in neonatal serum, maternal PAHs, neonatal PAHs of different delivered months were plotted on clustered bar charts and Pearson correlation coefficients were calculated. The annual variation curves for the AQI, PM_2.5_, PM_10_, SO_2_, NO_2_, and CO in Urumqi were plotted. Two air pollutants (PM_10_, O_3_) with strong oxidative properties and weak collinearity in air pollution and the level of 8-OHdG in cord blood were selected to construct a multiple stepwise linear regression model; Based on the epidemiological questionnaire, a multiple linear regression model was established. Factors affecting the exposure of pregnant women to PAHs were included in the study to explore their effects on 8-OHdG levels in in neonatal umbilical cord blood. All statistical analyses were conducted using SPSS, Version 22.0 (IBM, Armonk, NY, USA). A two‐tailed P < 0.05 was considered statistically significant.

## Results

In this study, 200 pregnant women and their newborns were enrolled. According to whether the delivery date of pregnant women was in the heating season period, the subjects were divided into two groups, one was the heating season group, the other was the non-heating season group, each group included 100 pairs of pregnant women and their newborns. Compared to the non-heating season group, the total content of 16 PAHs in maternal venous blood and neonatal umbilical cord blood was significantly higher (all P < 0.001) in the heating season group (Table 2). The 8-OHdG level in maternal venous blood and urine, and neonatal umbilical cord blood was also significantly higher (all P < 0.001) in a heating season group (Table 2).

**Table 2:** Baseline characteristics in pregnant women and their newborns

	Heating season (n,%;mean ± SD)	Non-heating season (n,%;mean ± SD)	*P*
Maternal Age(year)			0.285
15-	7(7.00)	12(12.00)	
25-	89(89.00)	81(81.00)	
35–44	4(4.00)	7(7.00)	
Gestation length (day)	275.06 ± 0.716	276.82 ± 0.654	> 0.05
Nationality			0.188
Han	95(95.00)	85(85.00)	
Minority	5(5.00)	15(15.00)	
AQI	106.91 ± 0.13	97.44 ± 0.95	< 0.001^*^
PM_2.5_(µg·m^− 3^)	65.72 ± 0.15	57.28 ± 0.92	< 0.001^*^
PM_10_(µg·m^− 3^)	116.91 ± 0.15	98.88 ± 1.49	< 0.001^*^
SO_2_(µg·m^− 3^)	9.03 ± 0.01	7.87 ± 0.03	< 0.001^*^
NO_2_(µg·m^− 3^)	46.37 ± 0.06	42.22 ± 0.33	< 0.001^*^
CO(µg·m^− 3^)	1.29 ± 0.01	1.14 ± 0.01	< 0.001^*^
O_3_(µg·m^− 3^)	46.94 ± 0.15	53.36 ± 0.75	< 0.001^*^
PAH(µg·L^− 1^)			
Maternal PAHs	17.57 ± 4.53	14.50 ± 4.65	< 0.001^*^
Newborn PAHs	14.10 ± 3.23	11.48 ± 3.28	< 0.001^*^
Concentration of 8-OHdG(mg·moL^− 1^)			
8-OHdG in maternal venous blood	2532.71 ± 299.19	2399.04 ± 284.18	0.001^*^
8-OHdG in maternal urine	1454.58 ± 306.15	600.32 ± 202.89	< 0.001^*^
8-OHdG in neonatal umbilical cord blood	2434.88 ± 299.89	1288.58 ± 766.93	< 0.001^*^

Note: * *P <* 0.05

It was found that maternal and neonatal PAHs were correlated with 8-OHdG in maternal urine (r = 0.288, P < 0.001 and r = 0.336, P < 0.001) and neonatal umbilical cord blood (r = 0.296, P < 0.001 and r = 0.252, P < 0.001) (Table 3).

**Table 3:** The correlation between PAHs exposure and 8-OHdG level in pregnant women and their newborns

	8-OHdG in maternal venous blood		8-OHdG in maternal urine		8-OHdG in neonatal umbilical cord blood	
	r	p	r	p	r	p
Maternal PAHs	0.095	0.181	0.288	< 0.001	0.296	< 0.001
Neonatal PAHs	0.125	0.077	0.336	< 0.001	0.252	< 0.001

**Fig. 2:**
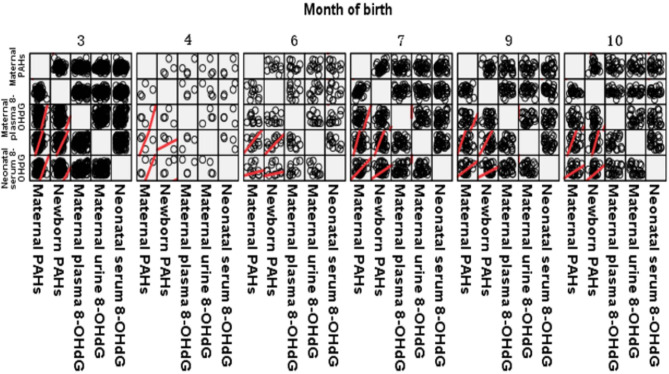
The relation between total PAHs levels and 8-OHdG levels in different birth months

**Fig. 3:**
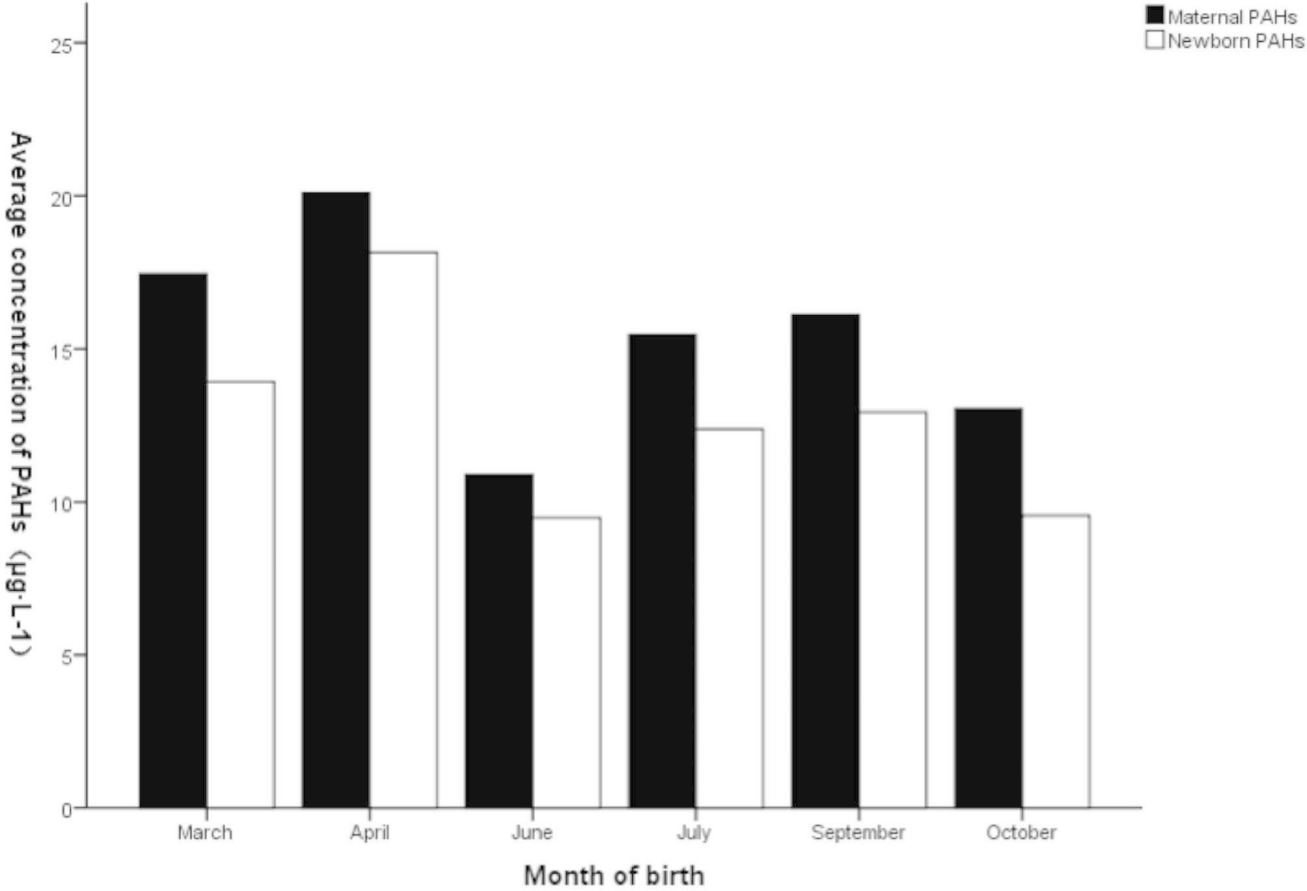
The total PAHs levels of pregnant women and neonates by birth month

**Fig. 4:**
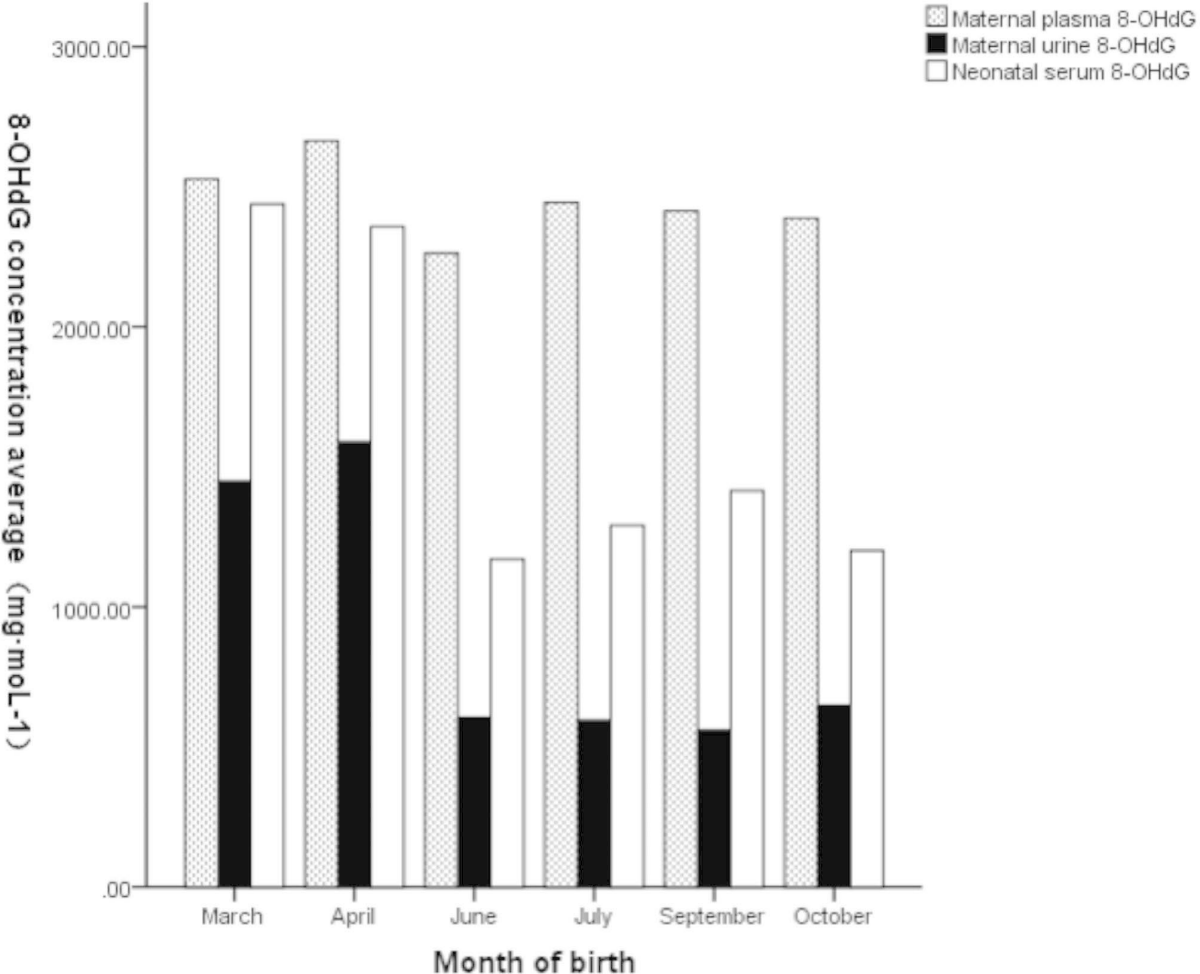
Maternal and neonatal 8-OHdG levels by birth month

The average value of PAHs at delivery was designated as the internal exposure dose of PAHs in pregnant women for that month. The internal exposure dose was significantly correlated with the 8-OHdG level in neonatal umbilical cord blood (Fig. 2). The levels of 8-OHdG in maternal venous blood, urine, and neonatal umbilical cord blood, as well as maternal and neonatal PAHs levels, are plotted on clustered bar charts by delivery month. These clustered bar charts demonstrate increased 8-OHdG and PAHs levels during seasons of high pollution exposure (Figs. 3 and 4, **and** Table 4).

**Table 4:** The trend of PAHs and 8-OHdG in pregnant women and newborns in different months (mean ± SD)

Month of birth	March (n = 71)	April (n = 4)	June (n = 19)	July (n = 45)	September (n = 36)	October (n = 25)
Maternal PAHs(µg·L^− 1^)	17.46 ± 4.38	20.10 ± 7.71	10.89 ± 1.56	15.46 ± 5.37	16.13 ± 4.23	13.05 ± 3.71
Newborn PAHs(µg·L^− 1^)	13.93 ± 3.18	18.16 ± 1.29	9.49 ± 1.70	12.38 ± 3.33	12.93 ± 2.57	9.56 ± 3.25
8-OHdG in maternal venous blood (mg·moL^− 1^)	2527.26 ± 301.07	2663.51 ± 244.80	2263.08 ± 298.52	2443.22 ± 279.33	2412.59 ± 272.92	2387.25 ± 292.41
8-OHdG in maternal urine(mg·moL^− 1^)	1448.98 ± 306.08	1588.82 ± 318.23	606.14 ± 151.94	595.57 ± 198.21	559.99 ± 221.81	649.34 ± 209.20
8-OHdG in neonatal umbilical cord blood (mg·moL^− 1^)	2438.06 ± 296.88	2358.37 ± 410.60	1169.47 ± 880.29	1290.99 ± 741.13	1414.68 ± 748.73	1201.12 ± 792.87

**Fig. 5:**
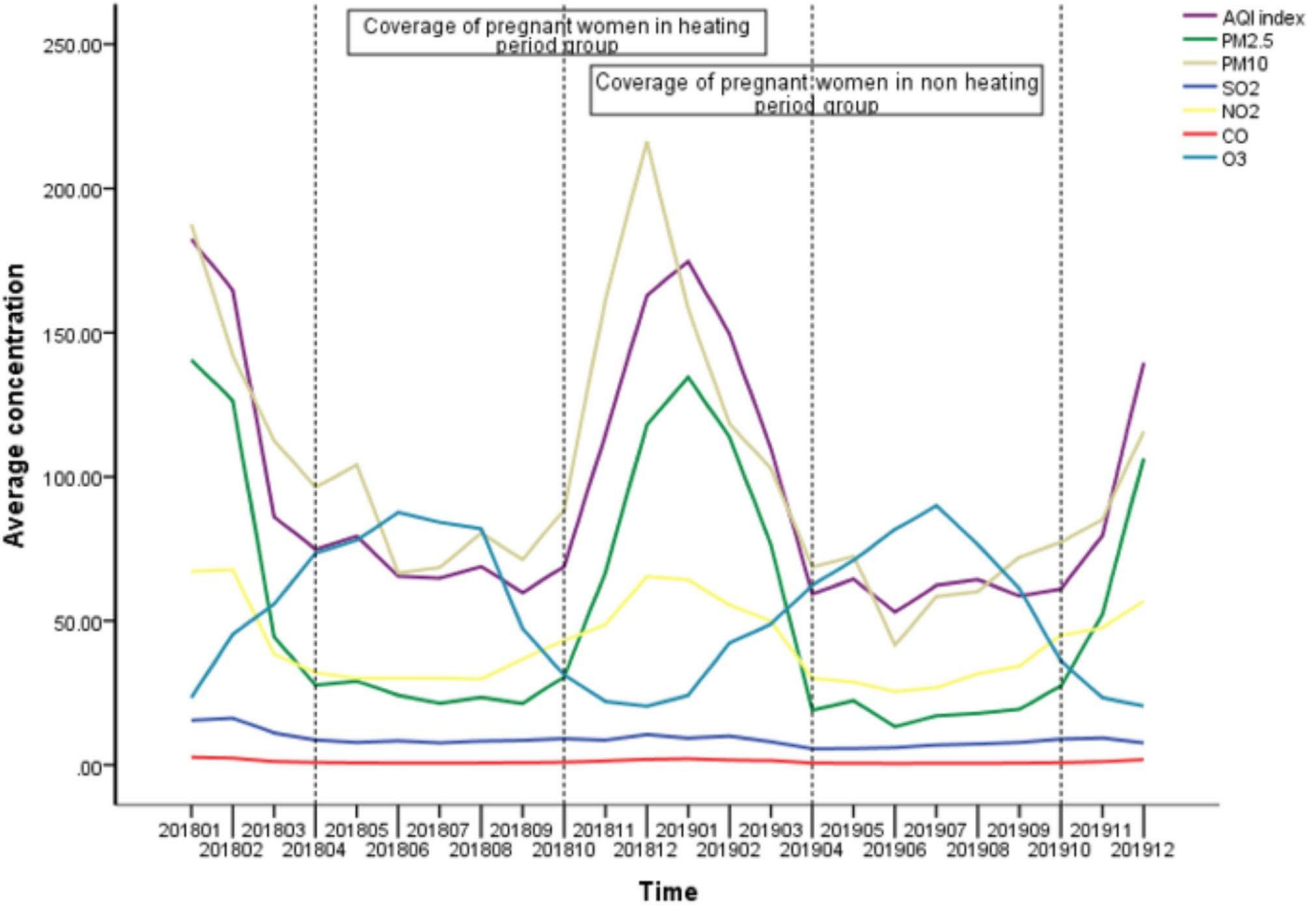
Air pollutant concentrations in Urumqi from January 2018 to October 2019

During the heating season, AQI, PM_2.5_, PM_10_, SO_2_, NO_2_, and CO were significantly higher than the non-heating season (all P < 0.001) (Table 2). The primary source of air pollution in Urumqi from January 2018 to December 2019 was from PM_10,_ PM_2.5_. Most air pollutants including PM_2.5_, PM_10_, and NO_2_ followed the same trend over time, except O_3_, which decreased during the heating season. The concentrations of SO_2_ and CO were relatively stable and did not fluctuate with time (Fig. 5). There was a significant correlation between PAHs, 8-OHdG levels in pregnant women and newborns and the concentration of air pollutants (AQI, PM_2.5_, PM_10_, SO_2_, NO_2_, CO, O_3_) (all P *<* 0.05). O_3_ was negatively correlated with PAHs and 8-OHdG levels in pregnant women and their newborns (all P < 0.05) (Table 5).

**Table 5:** The correlation between air pollutant concentration with maternal and neonatal PAHs, maternal and neonatal 8-OHdG levels

	AQI	PM_2.5_	PM_10_	SO_2_	NO_2_	CO	O_3_
Maternal ΣPAHs	0.196^*^	0.181^*^	0.226^*^	0.301^*^	0.220^*^	0.199^*^	-0.171^*^
Newborn ΣPAHs	0.364^*^	0.358^*^	0.329^*^	0.389^*^	0.359^*^	0.364^*^	-0.275^*^
8-OHdG in maternal venous blood	0.131	0.128	0.142^*^	0.192^*^	0.147^*^	0.146^*^	-0.123
8-OHdG in maternal urine	0.471^*^	0.444^*^	0.542^*^	0.793^*^	0.553^*^	0.508^*^	-0.441^*^
8-OHdG in neonatal umbilical cord blood	0.409^*^	0.385^*^	0.454^*^	0.645^*^	0.453^*^	0.430^*^	-0.351^*^

Note: * *P <* 0.05.ΣPAHs represents the total content of 16 PAHs. 1: Naphthalene; 2: Acenaphthylene; 3: Acenaphthene; 4: Fluorene; 5: Phenanthrene; 6: Anthracene; 7: Fluoranthene; 8: Pyrene; 9: Benzo[a ]pyrene; 10: Chrysene; 11: Benzo [g, h, i] peryleme; 12: Benzo[a]anthracene; 13: Benzo [b] fluaranthene; 14: Benzo [k] fluaranthene; 15: Indeno [1,2 ,3-cd] pyrene; 16: Dibenz [a, h] anthracene

Because these air pollutant levels (including: AQI, PM_2.5_, PM_10_, SO_2_, NO_2_, and CO) have certain collinearity, two types of air pollution(PM_10_, O_3_) with strong oxidizing and weak collinearity were screened. The amount of 8-OHdG in neonatal cord blood is represented by Y. A multivariate stepwise linear regression model was constructed for the levels of air pollutants (PM_10_, O_3_) and 8-OHdG in neonatal umbilical cord blood: Y=-18279.587 + 104.355*PM_**10**_ + 176.748*O_**3**_ ,**R**^**2**^ **= 0.320.**

Based on the epidemiological questionnaire, a multiple linear regression model was established. Factors affecting the exposure of pregnant women to PAHs were included in the study to explore their effects on 8-OHdG levels in neonatal umbilical cord blood. The amount of 8-OHdG in neonatal cord blood is represented by Y ,Y = 697.996 + 1122.219*X_**4**_-166.281*X_**12**_,**R**^**2**^ **= 0.496(** Tables 1 and 6).

**Table 6:** Pregnant women’s personal situation, life and surrounding environmental conditions during pregnancy

Index		Number of cases	Proportion(%)	
Pregnant women’s age	18-	1	0.5	
	25-	184	92.0	
	35-	15	7.5	
Pregnant women’s degree	Primary school and below	3	1.5	
	Middle school	31	15.5	
	College or university	166	83.0	
Number of delivery of pregnant women (including this time)	1	131	65.5	
	2	63	31.5	
	3	6	3.0	
Biological sample collection period	Non -heating period	100	50.0	
	Heating period	100	50.0	
Whether the housing is decorated within one year before childbirth	No	180	90.0	
	Yes	20	10.0	
The ventilation of the house where the house lives during pregnancy	Very good	151	75.5	
	Good	49	24.5	
	Poor	0	0	
The distance between the place of residence is from the road	Beside the road(≤400 m)	47	23.5	
	Closer(400-800 m)	132	66.0	
	Far away(≥800 m)	21	10.5	
Pregnancy heating method	Heating	196	98.0	
	Stove	3	1.5	
	Electricity	1	0.5	
Whether there are tapestry in housing during pregnancy	No	171	85.5	
	Yes	29	14.5	
Eat barbecue and fried food during pregnancy	No	183	91.5	
	Yes	17	8.5	
Husband smoke	No	137	68.5	
	Yes	63	31.5	
Gender of Newborn	Male	102	51.0	
	Female	98	49.0	

## Discussion

The results of previous studies have shown that atmospheric particulate matter pollution is serious during the central heating period in Urumqi in winter, atmospheric particulate matter is an important source of PAHs[1–4], and there is a correlation between PAHs and 8-OHdG concentration levels[20–21]. The study suggested that in the heating season, there are significantly higher levels of PAHs in maternal venous blood and neonatal umbilical cord blood, and 8-OHdG in maternal venous blood, urine and neonatal umbilical cord blood. It is suggested that exposure to PAHs during pregnancy can affect the levels of 8-OHdG in pregnant women and their newborns due to air pollutants.

PAHs can be found in fine PM and are known to induce cellular oxidative stress [22]. Others have reported a positive association between both PM_2.5_ and PAHs exposure and urinary 8-OHdG levels [23, 24]. Similarly, our findings indicate that PAHs exposure in pregnant women is correlated with maternal and neonatal 8-OHdG concentrations. Animals exposed to PAHs and motor vehicle exhaust have decreased antioxidant enzyme activity and increased levels of the oxidation product 8-OHdG [25]. When the scavenging capacity of the body is exceeded, oxidative stress can be induced, resulting in cellular and DNA oxidative damage [26].

From January 2018 to December 2019 (Time frame including gestation period, Fig. 5), the concentration of air pollutants in Urumqi varied with the season. The concentration of air pollutants was generally higher in winter and spring than in other times. The sole exception was ozone, which was highest during the summer months. This study found that the degree of air pollutants (AQI, PM_2.5_, PM_10_, SO_2_, NO_2_ and CO) were positively correlated with the PAHs exposure levels and the levels of 8-OHdG in pregnant women and their newborns. In winter and spring season, temperature is low and ultraviolet light is weak, especially when the concentration of particulate matter is high, atmospheric visibility is reduced, so the ultraviolet light is reduced, resulting in the less ozone production. There is a negative correlation between particulate matter and ozone. Although ozone is a known oxidant [27], ozone levels were negatively correlated with maternal and neonatal PAHs exposure level and 8-OHdG level, indicating that PM has a greater health impact than ozone in this region. Based on the results of multiple linear regression analysis, We know that both PM_10_ and O_3_ can affect cord blood 8-OHdG level, but in the equation, PM_10_ contributes more to cord blood 8-OHdG level, Possible explanations include that ozone is less able to cause oxidative damage as compared to atmospheric PM and PAHs in this region.

PAHs are widespread pollutants in environmental media such as the air, working environment, food, and drinking water. PAHs exposure in urban residents mostly comes from atmospheric fine particles and automobile exhaust [28]. Food can also be a source of PAHs exposure. PAHs can be produced during the processing of air-dried and smoked food and high-temperature cooking (barbecue, baking and frying) [29]. Diet is usually the main source of exposure to PAHs in people without occupational exposure and non-smokers [30]. This study found that the concentration of polycyclic aromatic hydrocarbons in pregnant women was positively correlated with the concentration of air pollutants (AQI, PM_2.5_, PM_10_, SO_2_, NO_2_ and CO). Based on the results of multiple linear regression analysis, it was found that air pollutants(PM_10_, O_3_) had a great influence on the level of 8-OHdG in neonatal cord blood, and the contribution rate was high.Therefore, it can be considered that air pollution is a significant source of PAHs exposure in pregnant women in Urumqi.

Based on the epidemiological questionnaire, a multiple linear regression model was established. We found that 8-OHdG levels in neonatal umbilical cord blood were mainly affected by two aspects: 1. Biological samples collected during heating had higher levels of 8-OHdG in neonatal umbilical cord blood. Combined with the above, we speculate that the possible reason is that biological samples from different sampling periods experience different levels of atmospheric pollutants during pregnancy;2. Study may suggest that in neonates, males are more sensitive to oxidative damage.Animal studies have shown that prepubertal animals are more susceptible to the effects of genotoxic estrogen, and males are more sensitive than females[31].Many studies have shown that women have lower oxidative DNA damage than men at different ages. Compared with men of the same age, women from birth to menopause have less DNA oxidative damage, and women have a biological advantage[32–34].However, these findings are mostly conclusions drawn in the adult group, and this study may suggest that in neonates, males are more sensitive to oxidative damage. Prenatal exposure to air pollutants should be paid more attention.

A study have shown that organic pollutants such as PAHs can freely pass through the placental barrier [35]. This study suggests that PAHs may penetrate the placental barrier and cause oxidative damage to the fetus, leading to elevated levels of 8-OHdG in umbilical cord blood. Since most persistent organic environmental pollutants are fat-soluble, when pregnant women lactate after delivery, the organic pollutants accumulated in the pregnant mother will be transferred to the infant through milk which will cause persistent harm to the development of infant [36]. Birth cohort studies have shown that exposure to PAHs during pregnancy can adversely affect the early development of 12-month-old children and the neurobehavioral development of 2-year-old children, resulting in reduced developmental levels or even developmental impairments, and may even increase their disease risk in adulthood[37–38].

The present study conclude that relevant measures should be taken at all stages of pregnancy until the end of lactation to avoid exposure to high concentrations of air pollutants. Fit-tested masks and air purifiers can help women to limit their exposure to air pollution in pregnancy. Moreover, diet and cooking methods should be monitored to reduce multichannel exposure to PAHs. The effects of PAHs exposure at different stages of pregnancy on offspring need to be further followed up.

There are some limitations of this study. The primary limitation of this study is that we can only conclude that proximate exposure to pollutants at birth cause higher levels of PAHs and 8-OHdG in umbilical cord blood. The study did not consider short-term and long-term variability in urinary PAHs concentrations. Second, there are many individual differences among pregnant, such as body weight that can affect the impact of a particular pollutant exposure level, quality control regarding PAHs and 8-OHdG measurements were not provided. Therefore, further analysis was needed. Third, the sample size of this study was limited.

## Conclusion

In conclusion, when pregnant women are exposed to air pollution, harmful substances such as PAHs can pass through the placenta. 8-OHdG is a marker of oxidative damage, and its level increases with PAHs exposure level. Pregnant women should monitor and control their exposure to air pollution during pregnancy, especially in the first trimester.

## Data Availability

All data generated or analysed during this study are included in this published article.
